# Creation and validation of a treatment algorithm for skeletally immature patients with acute anterior cruciate ligament injury based on MRI and patient characteristics

**DOI:** 10.1002/jeo2.70280

**Published:** 2025-08-05

**Authors:** Alberto Grassi, Kyle Borque, Martijn Dietvorst, Emanuele Altovino, Claudio Rossi, Luca Ambrosini, Alice Bondi, Stefano Zaffagnini

**Affiliations:** ^1^ Dipartimento di Scienze Biomediche e Neuromotorie DIBINEM Università di Bologna Bologna Italy; ^2^ Clinica Ortopedica e Traumatologica II, Istituto Ortopedico Rizzoli Bologna Italy; ^3^ Houston Methodist Hospital Houston Texas USA; ^4^ Máxima MC Eindhoven the Netherlands; ^5^ Dipartimento Rizzoli Sicilia, Istituto Ortopedico Rizzoli Bagheria Palermo Italy

**Keywords:** ACL, algorithm, MRI, paediatric, physeal sparing, skeletally immature

## Abstract

**Purpose:**

This study aimed to develop and validate a clinical decision‐making algorithm, the ‘Best ACL‐treatment Based on the Years of the Knee’ (BABY‐Knee) Algorithm, for treating acute anterior cruciate ligament (ACL) injuries in skeletally immature patients. The algorithm integrates magnetic resonance imaging (MRI) findings and patient‐specific characteristics to differentiate cases suitable for conservative management from those requiring surgical intervention.

**Methods:**

A prospective cohort of 75 skeletally immature patients (mean age: 13.9 ± 2.2 years) diagnosed with ACL rupture at a single institution between February 2022 and October 2024 was evaluated. Patients were categorized as surgical or non‐surgical candidates based on the BABY‐Knee Algorithm, which incorporates six weighted criteria: MRI‐detected meniscal tears, lateral tibiofemoral bone bruises, skeletal age, injury mechanism and rotatory laxity. Outcomes of initial management were retrospectively analyzed for algorithm validation.

**Results:**

Of the 75 patients, 55 (73.3%) underwent surgical reconstruction, while 20 (26.7%) were managed conservatively. Conservative treatment failed in 12 cases (60%), necessitating surgical intervention. Retrospective application of the algorithm yielded a positive predictive value of 91.7% for identifying surgical candidates and a negative predictive value of 87.5% for successful conservative treatment.

**Conclusion:**

The BABY‐Knee Algorithm demonstrated high reliability in guiding treatment decisions for skeletally immature patients with acute ACL injuries, predicting outcomes of conservative treatment in nearly 90% of cases. Further studies are required to confirm its applicability in additional prospective case series.

**Level of Evidence:**

Level IV, case series.

**Abbreviations**
ACL: anterior cruciate ligament
BABY‐Knee: Best ACL treatment Based on Years of the Knee
IOC: International Olympic Committee
MRI: magnetic resonance imaging
NPV: negative predictive value
PPV: positive predictive value

## INTRODUCTION

Although anterior cruciate ligament (ACL) tears in young and skeletally immature patients account for less than 5% of all ACL injuries [11, 26, 32], the absolute number has increased over recent years [2, 5, 31, 34]. Historically, these injuries were managed conservatively due to concerns about potential damage to the growth plates during surgical intervention [18], often leading to a restriction of sports participation until skeletal maturity [31]. However, this approach remains controversial due to two primary drawbacks. First, restricting sports participation can lead to psychological distress in children, causing feelings of isolation, frustration and depression [23]. Second, knee instability significantly increases the risk of subsequent meniscal injuries, with rates reaching as high as 60%–70%, particularly in younger patients [21, 33].

The International Olympic Committee (IOC) provided recommendations for guiding ACL treatment in skeletally immature patients, suggesting surgical intervention in three primary scenarios: (1) associated meniscal or cartilage lesions, (2) recurrent symptomatic knee instability and (3) unacceptable restrictions on sports or recreational activities. However, in the acute setting, recurrent instability episodes cannot be assessed as they typically manifest after an attempt at conservative treatment. Furthermore, meniscal and cartilage injuries are challenging to diagnose solely through imaging due to the limited sensitivity of magnetic resonance imaging (MRI) in identifying subtle lesions such as medial ramp and lateral root tears [20, 24]. As a result, clear guidelines for acute management remain lacking, and the decision to pursue surgery or conservative treatment continues to rely on individual clinician judgement.

The decision to operate on a skeletally immature patient with an ACL tear requires a multifactorial approach, considering various factors such as skeletal age, injury mechanism (contact vs. non‐contact), presence of concomitant meniscal and/or chondral lesions, severity of rotational laxity, type of sporting activity and the patient's desire to return to sports. Shared decision‐making between the surgeon, patient and parents/guardians is essential, yet no standardized framework exists to guide clinicians in presenting this information.

The present study aimed to develop and validate an algorithm to assist clinicians in determining the appropriate treatment for acute ACL injuries in skeletally immature patients. The hypothesis was that this algorithm would demonstrate acceptable reliability in distinguishing patients suitable for surgical versus conservative management.

## METHODS

All skeletally immature patients diagnosed with an ACL rupture and treated at Rizzoli Orthopedic Institute, Bologna, Italy, between February 2022 and October 2024 by a single high‐volume surgeon (A.G.) with experience in either adult or paediatric ACL reconstruction were prospectively enrolled in this study. Diagnosis was based on medical history, clinical examination and MRI assessment. Patients were classified as skeletally immature if an open physis was observed in the tibial tubercle, femur and/or tibia on knee MRI.

The decision between conservative management and ACL reconstruction was based on IOC guidelines and several other clinical parameters critically assessed by the treating surgeon, including skeletal age, presence of meniscal tears on preoperative MRI, presence of lateral tibiofemoral bone bruises, mechanism of injury (contact vs. non‐contact), sport and clinical assessment (rotatory laxity assessed with pivot‐shift testing).

Patients who initially underwent conservative treatment elsewhere were evaluated for recurrent instability episodes, pain or an inability to return to sports participation, which were considered indications for surgery.

For surgical cases, a physeal‐sparing ACL reconstruction technique with lateral tenodesis was performed [29]. Meniscal lesions were repaired with all‐inside sutures for radial, longitudinal, root and ramp tears and the posterior portion of bucket handle tears; out‐in sutures were used for the anterior portion of bucket handle tears. Ramp tears were explored with the trans notch view and probed with a spinal needle from the posteromedial portal.

Conservative treatment consisted of a structured rehabilitation programme lasting at least three months, following IOC guidelines. Return to sport was permitted only upon completion of rehabilitation, without episodes of pain, swelling or instability [1]. Treatment failure was defined as two or more giving‐way episodes, inability to return to the desired level of activity without instability, or evidence of a secondary meniscal tear on a follow‐up MRI performed at least 3 months post‐injury.

### MRI evaluation

The MRI scans of all patients were assessed by a single surgeon with expertise in ACL reconstruction and meniscal repair (A.G.). Meniscal lesions were identified based on specific imaging criteria. Longitudinal tears were diagnosed in the presence of a vertical hyperintense line within the meniscal tissue on T2‐weighted images. Bucket‐handle tears were recognized when a portion of the meniscus was displaced into the intra‐articular notch or in the presence of a ‘Double‐PCL Sign’. Root tears were defined by irregularities in the meniscal signal within 1 cm of the meniscofemoral attachment or the presence of a ‘ghost sign’ [24]. Ramp tears were identified by a hyperintense signal in the meniscal periphery or between the meniscus and the capsule, or in cases of bone marrow oedema in the posteromedial portion of the medial tibial plateau [20]. Finally, menisco‐tibio‐popliteus‐fibular complex lesions were diagnosed when the meniscus appeared intact but exhibited an interruption of the structures connecting it to surrounding anatomical features, as observed in either coronal or sagittal views [13].

Additionally, preoperative MRI was utilized to determine the patient's Bone Age according to Politzer et al. [28], based on the Pennock Atlas [27]. This assessment involved evaluating several MRI characteristics, including the status of the tibial and femoral physis, the ossification status of the tibial tubercle, and subchondral ossification, to estimate the patient's Bone Age. Given that inter‐ and intra‐rater reliability, as well as correlation with the Greulich and Pyle hand bone atlas, have been previously established [27, 28], these parameters were not reassessed in this study. The presence of the characteristic ACL‐related bone bruise pattern—localized to the anterior portion of the lateral femoral condyle and the posterior portion of the lateral tibial plateau [10]—was also evaluated.

### Patient evaluation

Clinically, anteroposterior, sagittal and rotatory laxities were manually assessed in an outpatient setting and graded from 0 to 3+ according to the objective International Knee Documentation Committee score [14]. Special attention was given to identifying ‘high rotatory laxity’, defined as the highest grade of pivot shift (Grade III or ‘gross’).

Furthermore, the mechanism of injury was investigated through discussions with patients and their families. Specifically, it was determined whether the injury resulted from a ‘contact’ or ‘non‐contact’ mechanism [9]. Additional inquiries included whether the injury occurred during the patient's preferred sport, whether it involved a common sports manoeuvre (e.g., pressing, change of direction and landing after a jump), and whether the patient intended to return to the same pre‐injury level of activity following ACL rupture.

### Algorithm creation

Based on the data acquired from this patient cohort, an algorithm was developed to standardize the decision‐making process between conservative and surgical treatment for ACL injuries in skeletally immature patients. This algorithm, named ‘Best ACL treatment Based on Years of the Knee’ (BABY‐Knee Algorithm), incorporated key variables considered by the first author in clinical decision‐making.

The algorithm consisted of three MRI‐based criteria and three patient characteristic criteria.

### MRI injury pattern

- Bucket‐handle or radial meniscal tears (3 points)
- Ramp or longitudinal meniscal tears (2 points)
- Lateral tibiofemoral bone bruises (1 point)

### Patient characteristics

- Skeletal age ≥13 years in males or ≥11 years in females (2 points)
- Non‐contact injuries occurring during the preferred sport (1 point)
- Grade 3 pivot shift (1 point)

### Algorithm Interpretation

The final algorithm score was obtained by summing the points associated with the identified criteria. The results were classified into two categories.

- Non‐Surgical Management (0–2 Points)
- Surgical Management (3–10 Points)

### Application of the algorithm

**Figure 1:**
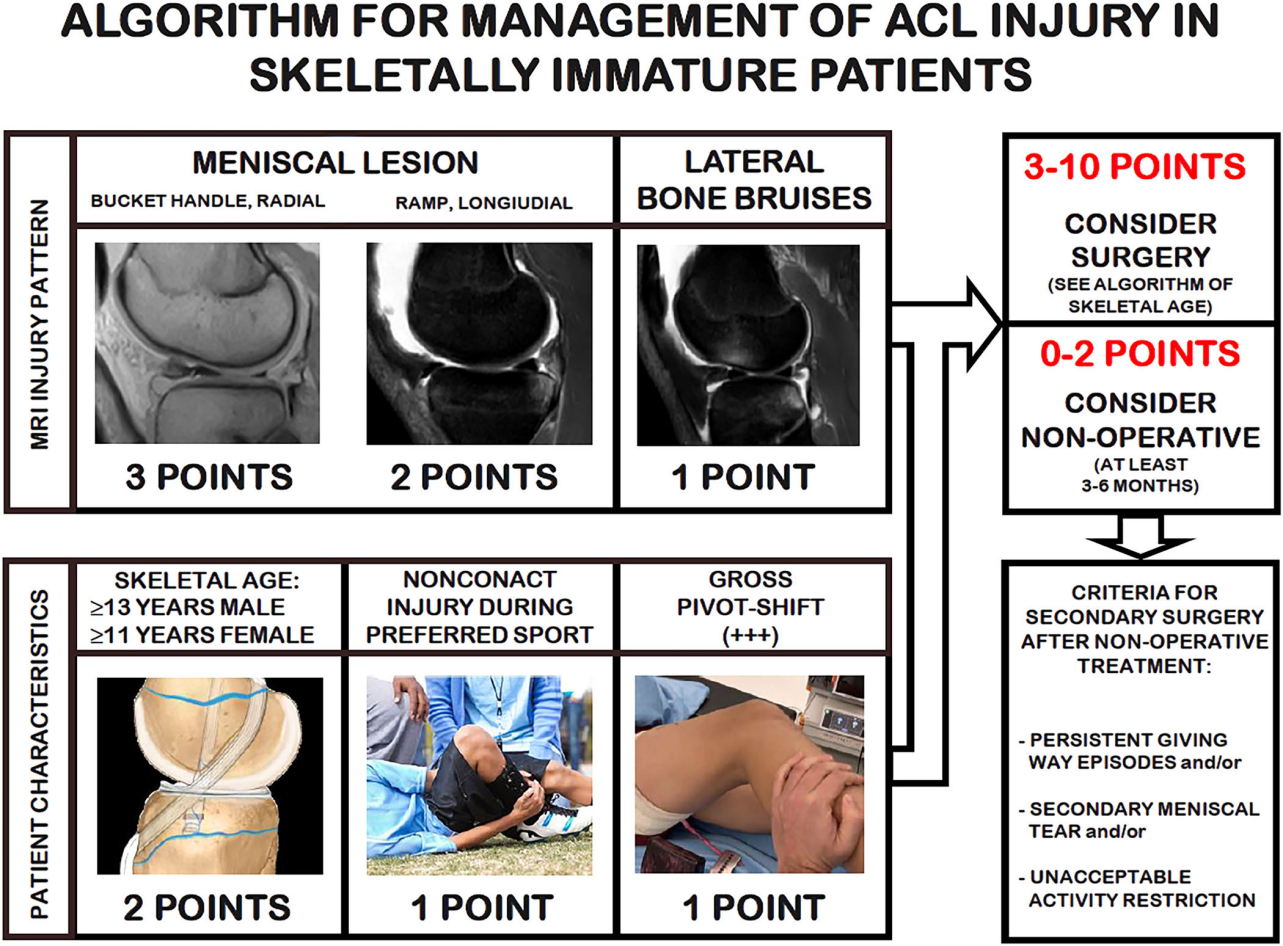
BABY‐Knee Algorithm. BABY‐Knee, Best ACL‐treatment Based on the Years of the Knee.

Following its creation, the algorithm was retrospectively applied to the same patient cohort to evaluate its alignment with the first author's management decisions. Cases in which patients had a score suggesting conservative management but were treated surgically were considered potential instances of overtreatment. Conversely, for patients managed conservatively, a minimum follow‐up of 3 months was required to assess potential conservative treatment failures and identify cases of undertreatment (Figure 1).

### Evaluation of the algorithm and statistical analysis

The algorithm's performance was assessed using positive predictive value (PPV) and negative predictive value (NPV). PPV was defined as the probability that a patient with a positive test result (score ≥3) would experience failure of conservative treatment. It was calculated as the number of true positives (patients with a score ≥3 who failed conservative treatment) divided by all patients with a score ≥3.

NPV was defined as the probability that a patient with a negative test result (score <3) would have a successful conservative treatment outcome. It was calculated as the number of true negatives (patients with a score <3 who did not fail conservative treatment) divided by all patients with a score <3.

Statistical analyses were performed using MedCalc (MedCalc Software). Continuous variables were reported as mean ± standard deviation, while categorical variables were presented as absolute numbers and proportions of the total sample. Independent sample *t* tests were used to compare continuous variables, and Fisher's exact test was applied to compare dichotomous categorical variables. Statistical significance was set at *p* < 0.05.

## RESULTS

### Initial patient management

A total of 75 skeletally immature patients (62 males, 13 females) with a mean age of 13.9 ± 2.2 years and ACL injuries were treated by the first author during the study period. Of these, 66 patients (88.0%) presented acutely following their first knee sprain, while 9 patients (12.0%) were referred for a second opinion after more than six months of conservative treatment following a documented ACL rupture.

Following clinical and MRI evaluation and discussion with parents, 11 patients (14.7%) were managed conservatively, while the remaining 64 patients (85.3%) underwent ACL reconstruction. For algorithm development, the nine patients who were assessed in the chronic setting (more than 6 months after conservative treatment) were classified as initially receiving non‐surgical treatment, and their score was calculated based on their status immediately post‐injury. In these cases, the Pivot‐Shift item was not assessed. Consequently, 20 patients (26.7%) were considered to have undergone conservative treatment, while 55 patients (73.3%) were considered to have received surgical treatment.

### Outcomes of conservative treatment

Among the 20 patients who initially received conservative treatment, 12 later underwent ACL reconstruction. The specific reasons for surgical intervention were as follows: four male athletes (20%—two footballers and two basketball players) successfully returned to play but developed a medial meniscus bucket‐handle tear; three male athletes (15%—two rugby players and one footballer) experienced giving‐way episodes upon returning to play after uneventful rehabilitation; one male footballer (5%) experienced multiple giving‐way episodes during rehabilitation; one female footballer and one female kickboxer (10%) reported pain, catching and locking symptoms, and instability during rehabilitation; one non‐active male patient (5%) had giving‐way episodes after completing rehabilitation. One male footballer (5%) was scheduled for surgery after experiencing three giving‐way episodes during rehabilitation.

In total, 12 out of 20 patients (60.0%) were classified as having failed conservative treatment.

### Outcomes of surgical treatment

Among the 64 patients who ultimately underwent ACL reconstruction, none required revision ACL surgery at an average follow‐up of 16.3 ± 8.1 months. There were no cases of knee extension loss or restriction in knee flexion. However, four patients (6.2%) required additional surgery on the ipsilateral knee: one patient (1.6%) underwent joint lavage for post‐operative haematoma, one patient (1.6%) underwent medial meniscectomy following failed meniscal repair and two patients (3.1%) required staple removal. Additionally, three patients (4.7%) underwent contralateral ACL reconstruction.

### Results of the algorithm

According to the retrospective evaluation of the algorithm, a total of 8 patients (10.6%) obtained a score of <3 points, while 67 patients (89.3%) received a score of ≥3 points. All 55 patients (100%) who were treated surgically had a score of ≥3 points, and thus, the score was consistent with the actual management. Differently, only 8 out of 20 patients (40.0%) who were treated conservatively had a score of <3 points (indicative of non‐operative management), while the other 12 had a score of ≥3 points (indicative of surgical treatment).

Regarding the predictive accuracy of the score, among the 12 patients with a score of ≥3 points who underwent conservative treatment, only one patient (8.3%) was not considered a failure. Therefore, the PPV of a score ≥3 in identifying patients who would fail conservative treatment (and thus require surgery) was 91.7%.

Among the eight patients with a score <3 points who underwent conservative treatment, only one patient (12.5%) was considered a failure, while the remaining seven patients (87.5%) successfully underwent conservative treatment for at least nine months. These patients returned to sport without limitations and had no giving‐way episodes (except for one case where the patient transitioned to a non‐pivoting sport to prevent instability). Thus, the NPV of a score <3 in identifying patients suitable for conservative management was 87.5%.

### Analysis of algorithm components

When comparing patients who underwent surgical versus conservative treatment, those undergoing ACL reconstruction were significantly older. However, other individual parameters did not show significant differences between the groups (Table 1). Among patients treated conservatively, those who failed treatment had a higher prevalence of meniscal tears. A similar pattern was observed in the subgroup analysis of 27 patients without preoperative indications of meniscal tears (Table 2).

**Table 1:** Comparison of the BABY‐Knee Algorithm characteristics based on ACL treatment in the whole patient cohort.

Item	Total (*n* = 75)	Surgery (*n* = 55)	Conservative (*n* = 20)	*p* **value**	Conservative fail: Yes (*n* = 12)	Conservative fail: No (*n* = 8)	*p* **value**	*p* Surg vs. Failed conservative
Meniscus tear: Bucket‐handle or radial tear	5 (6.6%)	5 (9.1%)	0 (0.0%)	*p* = 0.3146	0 (0.0%)	0 (0.0%)	*p* = 1.0000	*p* = 0.5775
Meniscus tear: Ramp or longitudinal tear	43 (57.3%)	35 (63.6%)	8 (40.0%)	*p* = 0.1048	8 (66.6%)	0 (0.0%)	*p* = 0.0128*	*p* = 1.0000
Lateral femoral and tibial bone bruises	57 (76.0%)	43 (78.1%)	14 (70.0%)	*p* = 0.3652	9 (75.0%)	5 (62.5%)	*p* = 0.6594	*p* = 0.6963
Skeletal age: ≥12 years males, ≥10 years females	60 (80.0%)	51 (92.7%)	9 (45.0%)	*p* = 0.0001*	8 (66.6%)	1 (12.5%)	*p* = 0.0197*	*p* = 0.0905
Non‐contact injury during preferred sport	65 (86.7%)	47 (85.4%)	18 (90.0%)	*p* = 1.0000	11 (91.6%)	7 (87.5%)	*p* = 1.0000	*p* = 1.0000
3+ Pivot‐Shift	34 (45.3%)	27 (49.1%)	7 (35.0%)	*p* = 0.4331	4 (33.3%)	3 (37.5%)	*p* = 1.0000	*p* = 0.5259
Pivot and bone bruise and non‐contact	26 (34.6%)	22 (40.0%)	4 (20.0%)	*p* = 0.2576	4 (33.3%)	0 (0.0%)	*p* = 0.1031	*p* = 1.0000
Score ≥3 points	67 (89.3%)	55 (100%)	12 (60.0%)	*p* = 0.0007*	10 (90.9%)	1 (12.5%)	*p* = 0.0011*	*p* = 0.1746
	5.0	5.6	3.6		4.7	2.1		

Abbreviations: ACL, anterior cruciate ligament; BABY‐Knee, Best ACL‐treatment Based on the Years of the Knee.

* *p* < 0.05.

**Table 2:** Comparison of the BABY‐Knee Algorithm characteristics based on ACL treatment in patients with no meniscal injuries.

Item	Total (*n* = 27)	Surgery (*n* = 15)	Conservative (*n* = 12)	*p* **value**	Conservative fail: Yes (n = 4)	Conservative fail: No (n = 8)
Lateral femoral and tibial bone bruises	18 (66.7%)	10 (66.7%)	8 (66.7%)	*p* = 1.0000	3 (75.0%)	5 (62.5%)
Skeletal age: ≥12 years males, ≥10 years females	16 (59.3%)	13 (86.7%)	3 (25.0%)	*p* = 0.0019*	2 (50.0%)	1 (12.5%)
Non‐contact injury during preferred sport	24 (88.9%)	14 (93.3%)	10 (83.3%)	*p* = 0.5692	3 (75.0%)	7 (87.5%)
3+ Pivot‐Shift	11 (40.7%)	6 (40.0%)	5 (41.7%)	*p* = 1.0000	2 (50.0%)	3 (37.5%)
Pivot and bone bruise and non‐contact	7 (25.9%)	5 (33.3%)	2 (16.7%)	*p* = 0.4082	2 (50%)	0 (0.0%)
Score ≥3 points	19 (70.4%)	15 (100%)	4 (33.3%)	*p* = 0.0002*	3 (75.0%)	1 (12.5%)
Average points	3.2	3.9	2.4		3.0	2.1

Abbreviations: ACL, anterior cruciate ligament; BABY‐Knee, Best ACL‐treatment Based on the Years of the Knee.

* *p* < 0.05.

## DISCUSSION

The most important finding of the present study is that the proposed algorithm for managing skeletally immature patients with acute ACL injuries demonstrates strong predictive ability in distinguishing between those who would benefit from conservative treatment and those requiring surgical intervention. By integrating assessments of skeletal age, meniscal injuries, injury mechanism, rotatory laxity, and bone bruises, the algorithm accurately identified 91.7% of patients who would fail conservative management and 87.5% of those who would not require surgery.

Currently, no other structured algorithms exist for managing acute ACL injuries in skeletally immature patients, making it difficult to compare the predictive capabilities of the BABY‐Knee Algorithm. The most authoritative guidelines in this field have been provided by the IOC [1], which primarily focuses on the sub‐acute or chronic phase of ACL injuries, highlighting recurrent instability, restrictions in physical activity and secondary meniscal tears as primary surgical indications. However, the optimal management of acute‐phase injuries remains controversial. Additionally, these guidelines have not been validated on a well‐defined cohort of skeletally immature ACL‐injured patients to assess their effectiveness in guiding treatment decisions.

Given these considerations, the BABY‐Knee Algorithm was developed to address key factors present in the acute phase when treatment decisions are not based solely on patient‐reported instability but also on the risk of failure with conservative treatment. Consistent with the IOC guidelines, the algorithm places significant emphasis on meniscal injuries, particularly those identifiable on MRI and associated with poor prognoses, as a nearly absolute criterion for surgical intervention. The additional algorithm components were selected based on clinical experience, existing recommendations and available evidence.

A crucial source of validation for the BABY‐Knee Algorithm is the prospective study by Kocher et al. [17], which stratified failure risk following three months of non‐operative treatment in 45 children and adolescents with arthroscopically confirmed partial ACL tears. In this cohort, 14 patients (31%) eventually required ACL reconstruction within an average of 10 months post‐rehabilitation, leading to a 69% success rate for conservative management. Predictors of successful non‐operative treatment included lower rotatory laxity (anteromedial bundle injury and Grade A Pivot‐Shift), younger skeletal age (<14 years), and minor ACL lesions (>50% tissue remaining). These findings played a pivotal role in shaping the present algorithm.

Another influential study was conducted by Janarv et al. [15], which investigated the natural course of ACL rupture in 23 skeletally immature patients following a three‐month rehabilitation programme. Among these patients, 15 (65%) ultimately required ACL reconstruction. Key predictors for avoiding surgery included younger age (11.9 vs. 13.3 years) and a lower desired level of activity, reinforcing the role of age in sports participation and treatment selection.

### Explanation of algorithm components

To help the readers better understand how to interpret the algorithm's items and understand their importance, each of them is discussed separately.

#### Bucket handle or radial meniscal tears (3 points)

In this algorithm, these meniscal tears are considered absolute indications for surgery, requiring both meniscal repair and ACL reconstruction. Such tears significantly impact knee biomechanics [12], and delaying treatment may compromise repairability [13], potentially leading to meniscectomy and increased osteoarthritis risk, particularly in active young patients [12]. Additionally, untreated bucket‐handle and radial tears can cause disabling symptoms, including pain, locking, and progressive instability. Their reliable MRI diagnosis [25] further supports their prioritization in the algorithm.

#### Ramp or longitudinal meniscal tears (2 points)

These tears have been assigned a lower weight in the algorithm due to their comparatively lesser biomechanical impact, milder symptoms and reduced short‐term progression risk [22]. Furthermore, the diagnostic reliability of MRI for Ramp and Longitudinal tears is suboptimal [20], especially for smaller lesions, making them unsuitable as stand‐alone surgical criteria. Root tears were excluded from the algorithm due to their diagnostic challenges on MRI [24]; however, certain types (LaPrade Types III and IV) may be classified as radial or longitudinal tears on imaging [8].

#### Lateral tibiofemoral bone bruises (1 point)

The presence of bone bruises on the anterior/central lateral femoral condyle and posterior lateral tibial plateau is a hallmark of ACL injuries. When ligament continuity on MRI is inconclusive, this pattern serves as a reliable indirect indicator of ACL rupture. A study on 74 skeletally immature patients with lateral tibiofemoral bone bruises found that only 11% had an intact ACL [3]. Moreover, this characteristic bone bruise pattern is frequently associated with non‐contact injuries during directional changes in footballers [10], making it a surrogate marker for rotatory instability. However, caution is warranted in non‐acute settings, as bone oedema presence and intensity may vary depending on the time elapsed since injury.

#### Skeletal age ≥13 years for males or ≥11 years for females (2 points)

Older skeletal age has been identified as a risk factor for conservative treatment failure in children with partial and complete ACL tears [15, 17], likely due to increased knee stiffness [4, 30]. This reduced ligamentous laxity tolerance suggests that early surgical intervention may be more appropriate. Furthermore, adolescent patients typically present fewer technical challenges for ACL reconstruction and have limited remaining knee growth (5–1 cm) [16], minimizing the clinical significance of potential growth disturbances [7]. Consequently, chronological age is included in the algorithm despite not being a stand‐alone criterion for surgery.

#### Non‐contact injuries during preferred sport (1 point)

Given that most ACL injuries in skeletally immature patients occur in young athletes [32], the patient's desire to return to sports is a critical factor. If an ACL injury occurs through a non‐contact mechanism during a typical sports movement (e.g., deceleration, directional change and landing), there is a high likelihood of recurrent instability upon returning to the same sport. Conversely, a sedentary child with an ACL injury sustained through a contact mechanism or an atypical incident (e.g., fall and accident) may be more suitable for conservative management. This context has been incorporated into the algorithm.

#### Gross Pivot‐Shift (+++) (1 point)

**Figure 2:**
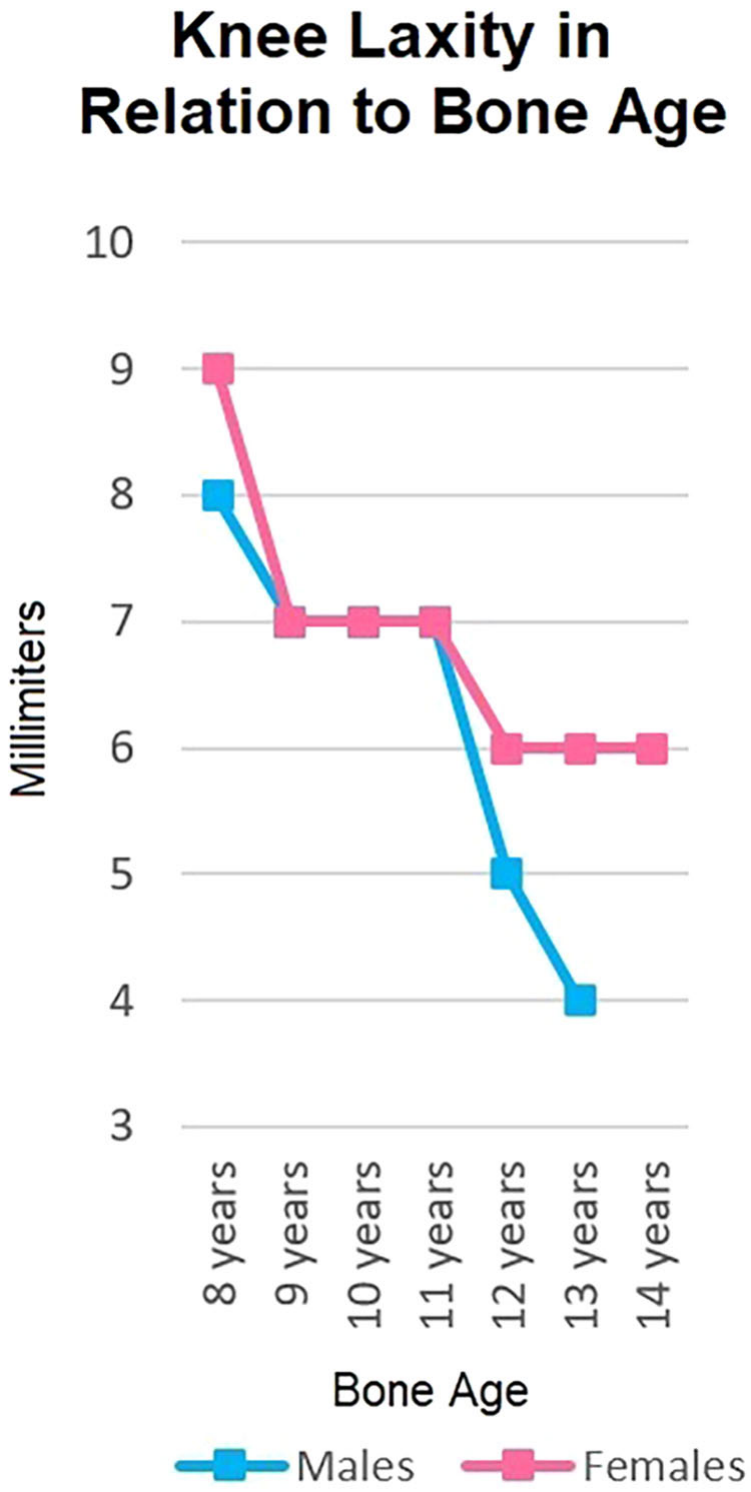
Knee joint laxity in males (blue line) and females (pink line) according to age, showing a progressive decrease of laxity with growth (Chart adapted from data by Baxter [4] and Seil [30] et al.).

A gross Pivot‐Shift indicates significant knee laxity and correlates with symptomatic instability [19]. Participating in sports with a highly unstable knee increases the risk of additional sprains and overloading secondary stabilizers [6], potentially leading to meniscal injuries. Although manual knee laxity assessment can be challenging and influenced by skeletally immature patients' inherent hyperlaxity (Figure 2), only the highest degree (3+) is considered a minor surgical indication.

### Algorithm interpretation

The final score of the six‐item algorithm is obtained by simply adding the points corresponding to the fulfilled items. The result is dichotomized into two scenarios.

#### Non‐surgical management (0–2 points)

This scenario could include ‘minor’ meniscal lesions or *Young/Old Adolescent* (2 points) patients without other items. Other possibilities are the combinations of no more than two 1‐point items. The presence of Bucket Handle or Radial tears automatically excludes the option of non‐operative management.

When the conservative treatment is chosen, it should be pursued for at least 3–6 months, carefully monitoring the patient to identify instability episodes or further subtle traumas. A new MRI is suggested after 3–6 months to identify possible secondary meniscal lesions and reassess skeletal age. The score should be calculated again considering possible changes in MRI, skeletal age, or laxity, and thus, the management should be reassessed accordingly. Other indications for surgery after conservative treatment are (1) persistent giving‐way episodes during rehabilitation, (2) the presence of a secondary meniscal tear and (3) unacceptable activity restriction to avoid instability episodes.

#### Surgical management (3–10 points)

This scenario could include the isolated presence of ‘major’ meniscal lesions (3 points) or the combination of other minor items. A low threshold for surgical treatment has been attributed to adolescents and older patients, for whom only a single 1‐point item is required to meet the surgical indication.

### Limitations

The present study has several limitations. First of all, the number of patients treated conservatively was lower compared to those treated with ACL reconstruction and this aspect could have reduced the accuracy of score. However, it was the approach of the authors to propose surgical or conservative treatment after a case‐by‐case specific assessment, instead of trying nonoperative treatment in all patients. Another limitation is that this study does not assess in detail the outcomes of surgical treatments and their risks; however, it was not the aim to compare the results and complications of the surgical and non‐surgical approach, since there is a great variety of surgical techniques, each with specific features and risks. In this regard, it should be stated that all patients who underwent surgical treatment had a score >3 points and thus could have been defined as sub‐optimal candidates for conservative treatment, due to the high risk of failure according to the algorithm properties. Moreover, the authors already performed a clinical study on ACL reconstruction in skeletally immature patients demonstrating satisfactory mid‐term outcomes and limited complications [29].

Finally, the threshold for surgery is low according to the score, which would make it uncommon to identify patients with a score < 3. However, these low‐score patients are generally those with isolated or partial tears and no risk factors for instability, which could thus be suitable for nonoperative management.

## CONCLUSION

The BABY‐Knee Algorithm demonstrated good predictivity in identifying skeletally immature patients who are suitable for initial conservative treatment after an acute ACL injury or who need immediate ACL reconstruction. However, the external validity of the algorithm and the longer‐term confirmation of its results should be confirmed in further studies.

## AUTHOR CONTRIBUTIONS

All authors contributed to the study conception and design. Material preparation, data collection and analysis were performed by Alberto Grassi, Kyle Borque and Luca Ambrosini. The first draft of the manuscript was written by Alberto Grassi, Martijn Dietvorst, Emanuele Altovino, Claudio Rossi and Stefano Zaffagnini. All authors commented on previous versions of the manuscript. All authors read and approved the final manuscript.

## CONFLICT OF INTEREST STATEMENT

Alberto Grassi: not‐paid consultant for Smith & Nephew. Kyle Borque: paid consultant for Mitek and Xiros Inc.; receives research support from CONMED Linvatec and Xiros Inc. Stefano Zaffagnini: paid consultant for De Puy Synthes and Smith & Nephew; Board member of European Society of Sports Traumatology Knee Surgery and Arthroscopy (ESSKA) and International Society of Arthroscopy, Knee Surgery, and Orthopaedic Sports Medicine (ISAKOS); Editor in Chief of Journal of Experimental Orthopaedics (JEO). The remaining authors declare no conflicts of interest.

## ETHICS STATEMENT

The study was conducted after approval by the local Ethical Committee (CE‐AVEC 380/2019/Oss/IOR, protocol number 0006881). The study was conducted in accordance with the Helsinki Declaration, and all patients gave written informed consent to participate. Parents provided written consent to include the patients in this study since all patients were minors (age < 18) at the time of participation.

## Data Availability

The DAS describes the availability of data.
